# Colistin use in pediatric intensive care unit for severe nosocomial infections: experience of an university hospital

**DOI:** 10.1186/1476-0711-12-32

**Published:** 2013-11-07

**Authors:** Arzu Karli, Muhammet Sukru Paksu, Adil Karadag, Nursen Belet, Sule Paksu, Akif Koray Guney, Muhammet Akgun, Nazik Yener, Sema Gulnar Sensoy

**Affiliations:** 1 Faculty of Medicine, Department of Pediatric Infection Disease, Ondokuz Mayis University, Samsun, Turkey; 2 Faculty of Medicine, Pediatric Intensive Care Unit, Ondokuz Mayis University, Samsun, Turkey; 3 Faculty of Medicine, Department of Clinical Microbiolgy, Ondokuz Mayis University, Samsun, Turkey; 4 Faculty of Medicine, Department of Pediatrics, Ondokuz Mayis University, Samsun, Turkey; 5 Department of Microbiology, Ataturk Chest Diseases and Chest Surgery Training and Research Hospital, Ankara, Turkey; 6 Ondokuz Mayis Universitesi Tıp Fakultesi Cocuk Hastanesi, 55139 Samsun, Turkey

**Keywords:** Colistin, Child, Multi-drug resistant bacteria, Nosocomial infection, Nephrotoxicity

## Abstract

**Background:**

The aim of this study was to investigate the efficacy and safety of colistin therapy in pediatric patients with severe nosocomial infections in pediatric intensive care unit.

**Methods:**

The medical records of patients treated with colistin at a 200-bed university children hospital were reviewed.

**Result:**

Thirty-one patients (male/female = 22/9; median age, 3 years; range, 3 months-17 years) received forty-one courses of colistin. The average dose of colistin was 4.9 ± 0.5 mg/kg/day and average treatment duration was 19.8 ± 10.3 days. Three patients who received concomitant nephrotoxic agent with colistin developed nephrotoxicity. Colistin treatment was well tolerated in other patients, and neurotoxicity was not seen in any patient. Favourable outcome was achieved in 28 (68.3%) episodes. Twelve patients died during the colistin therapy. Six of these patients died because of primary underlying disease. The infection-related mortality rate was found 14.6% in this study.

**Conclusion:**

In our study, colistin therapy was found to be acceptable treatment option for the severe pediatric nosocomial infections caused by multi-drug resistant bacteria. However, the use of concomitant nephrotoxic drugs with colistin must be avoided and renal function test should be closely monitored.

## Introduction

Colistin, an antibiotic with a bactericidal effect against gram-negative bacteria, was introduced for use in the 1950s. However, an abundance of side effects and the availability of safer alternatives have limited its use [1,2]. *Multi-drug resistance* (MDR) is defined as the resistance of microorganisms to at least three different groups of antibiotics with intrinsic activity against gram-negative bacteria [3]. Nosocomial infections caused by MDR microorganisms have been increasing with frequency and today are a common problem in all areas of the world [4-6]. The problem of resistance to existing drugs, and the slowing rate at which new drugs are being invented in this pharmacologic area, have caused reconsideration of colistin for use in this context [4,7-9]. Studies on the efficacy and safety of colistin therapy in children are rare [4,8,10-12]. This study investigated the efficacy and safety of colistin therapy in children with severe nosocomial infections in pediatric intensive care unit (PICU).

## Materials and methods

The study was performed at a PICU of 200-bed university children hospital. Forty-one nosocomial infection episodes that were treated with intravenous colistin and occurred in thirty-five children between March 2008 and March 2013 were analyzed retrospectively. Neonatal patients and patients who received colistin treatment for less than 4 hours were excluded from the study. Information recorded included clinical and demographic characteristics, medical history, presence of medical devices such as central catheters, urinary catheters, and endotracheal or tracheostomy tubes, concomitant drug use especially can effect neurological or renal systems, clinical and laboratory findings, type and antimicrobial susceptibility of the isolated microorganism, type of infection that required colistin treatment (bloodstream infection, ventilator-associated pneumonia, etc.), dose, administration route, duration and side effects of colistin, results of antimicrobial therapy, and prognosis. The levels of blood urea nitrogen (BUN), creatinine, C-reactive protein (CRP) and the counts of leukocytes and platelets were recorded (at the beginning and during treatment). Clinical and laboratory data were obtained from patients’ cards and from the hospital computer database system. Pediatric risk of mortality (PRISM) and pediatric logic organ dysfunction (PELOD) scores were used to determine the severity of illness and organ dysfunction respectively. The medical records of each patient were investigated also for signs of neurotoxicity (neuromuscular blockade, seizures, change in level of consciousness, etc.) during treatment.

The diagnosis of *infection* was made by clinical and laboratory findings and through positive culture. The diagnosis of *nosocomial infection* and specific infections such as *catheter-related infection*, *ventilator-associated pneumonia (VAP)* was made on the basis of the criteria of the Centers for Disease Control and Prevention [13]; sepsis was defined according to the definition of the International Pediatric Sepsis Consensus Conference [14].

Some of the important terms used in this paper were defined as follows: *Multi-drug resistance* was defined as the resistance of microorganisms to at least three different groups of antibiotics with intrinsic activity against gram-negative bacteria (e.g., beta-lactams, aminoglycosides, carbapenems, and quinolones) [3]. Empirical treatment was defined as the beginning of colistin treatment based on surveillance data, or on inadequate response of the infection to other antimicrobials [10]. *Response to treatment* was defined as improved clinical and laboratory findings of index infection, and eradication of the pathogen microorganism isolated by initial culture. If the patient was alive at the end of treatment, prognosis was evaluated as a survey. Patients who died during treatment were also assessed in order to find out if the death had been caused by infection. *Nephrotoxicity* was defined in patients with normal baseline renal functions as serum creatinine levels higher than >1.1 mg/dl, or by a >50% reduction in creatinine clearance or by the need for renal replacement treatment (RRT) at any time during treatment.

Descriptive statistics were used in the data analysis. The study was approved by the Ondokuz Mayis Universitiy Human Studies Ethics committee. (No:2011/2062)

## Results

In this study 41 nosocomial infection episodes treated by iv colistin in 35 pediatric patients were reviewed. Six patients had two episodes. Four patients who received colistin treatment for less than 48 hours were not included in the study. The median age of patients was 3 years (range, 3 month-17 years). Five (14.3%) patients had been previously healthy; the other patients had an underlying chronic disease. Underlying diseases, in order of frequency, were as follows; neurological/neuromuscular disorders (28.6%), congenital heart disease (22.9%), inherited metabolic disorders (14.3%), primary immune deficiency (11.4%), malignancy (2.9%) and others (5.7%). When colistin therapy started, all patients except for one were on ventilation. The clinical and demographic characteristics of study patients were shown in Table 1.

**Table 1:** Demographic and clinical characteristics of study patients

**Age (month) [median (minimum-maximum)]**		36 (3–204)
**Male gender [number (%)]**		22 (%62.9)
**Weight (kg) [median (minimum-maximum)]**		12 (3–70)
**Comorbidity [number (%)]**		30 (85.7%)
● Chronic neurological or neuromuscular disorders	10 (28.6%)	
● Congenital heart disease	8 (2.9%)	
● Inherited metabolic disorders	5 (14.3%)	
● Primary immune deficiency	4 (11.4%)	
● Malignancy	1 (2.9%)	
● Others	2 (5.7%)	
**Immune deficiency [number (%)]**		9 (%25.7)
● Primary immune deficiency	2	
● Immunosuppressive treatment	7	
**Cause of PICU admission**		
● Respiratory failure	21 (%60.0)	
● Acute loss of consciousness, CNS infections or status epilepticus	6 (%17.1)	
● Post-operative follow-up or complications	4 (%11.4)	
● Sepsis or shock	3 (%8.6)	
● Poisoning	1 (%2.9)	
**Length of stay in PICU before infection (day) [median (min-max)]**		30 (0–126)
**Total length of stay in hospital before infection (day) [median (min-max)]**		39 (0–142)
**Duration of mechanical ventilation (day) [median (min-max)]**		25 (0–108)
**Medical devices (existed at the beginning of colistin treatment)**		
● Endotracheal or tracheostomy tube	41 (100%)	
● Central venous or arterial catheter	33 (80.5%)	
● Foley catheter	30(73.2%)	
● Thorax tube	2 (4.9%)	
● External ventricular drainage catheter	2 (4.9%)	
● Peritoneal dialysis catheter	1 (2.4%)	
**PRISM score at admission of PICU**		18.4 ± 9.2
**PELOD score at the start of colistin treatment**		16.2 ± 9.7
**Organ failure at the start of colistin treatment**		
● Neurological	30 (73.2%)	
● Respiratory	40 (97.6%)	
● Cardiac	12 (29.3%)	
● Renal	3 (7.3%)	
● Hepatic	2 (4.9%)	
● Hematologic	None	

**PRISM:** Pediatric Risk of Mortality, **PELOD:** Pediatric Logistik Organ Dysfunction.

In 5 patients, intravenous colistin therapy was started empirically because of fever that was unresponsive to broad-spectrum antimicrobial treatment and progressive sepsis. In other patients, indication of colistin treatment was culture-proven infection. The most frequently isolated microorganism, the most common isolation site and type of infections were *A. baumannii*, tracheal aspirate, and VAP respectively.

The microorganisms were isolated from a single site in 58.3% of the culture-proven cases and from more than one site in 41.7%. All of the patients had received antimicrobial treatment in different combinations before and during colistin treatment. The drugs which effective against gram negative bacteria most frequently used with colistin were carbapenems and aminoglycosides in order of frequency. The properties of nosocomial infections treated by colistin and isolated microorganisms were shown in Table 2.

**Table 2:** Properties of nosocomial infections treated by colistin and causative microorganisms

**Indications of colistin treatment (%)]**	
● Culture-proven infection	36 (87.8%)
● Empirically	5 (12.2%)
**Causative microorganism [number (%)]**	
● *A. baumannii*	20 (48.8%)
● *P. aeruginosa*	9 (22.0%)
● *A. baumannii* and *P. aeruginosa*	7 (17.1%)
● No microorganism	5 (12.2%)
**Isolation sites of the microorganisms**	
● Tracheal aspirate fluid	24 (58.5%)
● Blood or central venous catheter tip	19 (46.3%)
● Skin swabs, conjunctival swabs	4 (9.8%)
● Cerebrospinal fluid	2 (4.9%)
**Concomitant antimicrobial agent effective against gram negatives used with colistin [number (%)]**	
● Carbapenems	22 (53.7%)
● Aminoglycosides	14 (34.1%)
● Piperacillin-tazobactam	5 (12.2%)
● Cefoperazone-sulbactam	1 (2.4%)

Colistin was administered intravenously in all patients; none of the patients received concomitant nebulized treatment. Only one patient had received intrathecal treatment in addition to iv route due to shunt infection. Due to impaired renal function, dosage adjustment was made in three patients at the beginning of treatment and in one patient during treatment. The average dose of colistin was 4.90 ± 0.5 mg/kg/day in patients without renal impairment, and considering all the episodes, the average duration of treatment was 19.8 ± 10.3 days (surviving patients 23.1 ± 10.0, non-surviving patients 11.8 ± 5.6). Dose, duration, and side effects of colistin, and treatment results were shown in Table 3.

**Table 3:** Dose, duration, and side effects of colistin, and treatment results

**Dose in patients without renal failure (mg/kg)**		4.90 ± 0.5
**Duration (day)**		19.8 ± 10.3 (4–62)
● Favorable outcome	23.1 ± 10.0 (8–62)	
● Poor outcome or discontinuation of therapy	11.8 ± 5.6 (4–22)	
**Side effects (renal function impairment)**		3 (7.3%)
**Using of sedative or analgesic agents during the colistin treatment**		28 (68.3%)
**Concomitants nephrotoxic agent use**		34 (82.9%)
**Common nephrotoxic agents used with colistin**		
● Glycopeptides	23 (56.1%)	
● Aminoglycosides	14 (34.1%)	
● Furosemide	11 (26.8%)	
● Amphotericin B	10 (24.4%)	
● Antiviral agent (acyclovir)	1 (2.4%)	
● Radiocontrast	1 (2.4%)	
**Result of treatment:**		
● **Survey**		29 (70.7%)
○ Clinical and microbiological response	28	
○ Discontinuation of treatment due to side effect	1	
● **Exitus**		12 (29.3%)
○ Infection related death	6	
○ Infection unrelated death	6	

Renal replacement therapy and dose-adjusted colistin were started in three patients who had renal insufficiency before the colistin therapy. One of these patients had chronic renal failure and was on dialysis treatment. The other two patients had renal impairment as a component of multiorgan failure. While a single hemodialysis session (P4) was performed in one of these patients, the other patient underwent peritoneal dialysis (P38). General characteristics of the patients with renal impairment at the beginning of or during therapy were given in Table 4.

**Table 4:** General characteristics of the patients with renal insufficiency at the beginning and in the course of treatment

**Patient number**	**Age & gender**	**Time of RI**	**Type of RI**	**BUN, creatinine at the beginning of treatment**	**BUN, creatinine at the end of treatment**	**Concomitants nephrotoxic agent use**	**Type and duration of RRT**	**Duration of treatment**	**Outcome**
**P4**	11/M	Before treatment	Oliguric ARF	36/1.3	23/1.6	Amphotericin B	One day of hemodialysis	19	Survey
**P8**	7/M	During the treatment	Oliguric ARF	8/0.2	53/5.6	Aminoglycoside, glycopeptides	14 days of peritoneal dialysis	22	Survey
**P12**	2.5/F	During the treatment	Non-oliguric ARF	7/0.2	20/1.6	Aminoglycoside	Colistin treatment was halted	8	Survey
							RRT was not performed		
**P15**	12/F	During the treatment	Non-oliguric ARF	7/0.2	89/3.2	Glycopeptides, amphotericin B	RRT was not performed	8	Death*
**P26**	17/M	Before treatment	CRF	66/2.5	111/2.5	Non	8 days of hemodialysis	10	Death*
**P38**	7/M	Before treatment	ARF	76/2.3	38/1.2	Glycopeptides	4 days of peritoneal dialysis	4	Death**

*Infection-related death, **Infection-unrelated death, **BUN:** Blood urea nitrogen, **RI:** Renal impairment, **RRT:** Renal replacement therapy, **ARF:** Acute renal failure, **CRF:** Chronic renal failure.

Only three of thirty-four episode used concomitant nephrotoxic agent with colistin developed renal impairment. All three of these patients received at least one nephrotoxic agent such as aminoglycosides, amphotericin B or a glycopeptides together with colistin. In patient 8, peritoneal dialysis was started after 13 days of colistin treatment because of oliguric renal failure and remained for 14 days. Gentamicin treatment was discontinued and doses of colistin were adjusted according to the creatinin clearance in this patient. Level of creatinine was 5.6 mg/dl at the end of 22 days of colistin treatment and returned to normal value 18 days after the end of treatment. Acute renal failure developed after eight days of treatment in patient 12. Colistin treatment was discontinued and bloodstream infection caused by *P. aeruginosa* was treated successfully with meropenem in this patient despite in-vitro resistance. In patient 15 who had ataxia telengiectasia, non-oliguric renal failure developed on the fourth day of colistin treatment due to severe sepsis and septic shock. Colistin was continued in this patient because of severe sepsis and immune deficiency. The patient died on the eighth day of treatment before RRT was performed. Possible cause of renal failure in this patient was refractory shock and multiorgan dysfunction syndrome. We did not find neurological side-effects (change of consciousness, seizures, visual disturbances, or neuromuscular blockade) in any patients. However, most of the patients were comatose or were receiving sedative-analgesic agent.

In twenty-eight (68.3%) episodes, positive clinical response was achieved. Twelve patients died while receiving colistin therapy. Six of these patients died because of infection-related reasons, and others died of primary underlying disease. Renal impairment was developed in one patient who died due to infection-related reasons; however, RRT was not performed due to sufficient urine output. Positive clinical and microbiological results were obtained in other patients (Table 3).

## Discussion

Nosocomial infections caused by multidrug-resistant gram-negative microorganisms are an important cause of morbidity and mortality in intensive care units [15,16]. We previously reported the results of a multicenter study investigating the efficacy and safety of colistin treatment in PICU [12]. In this study we aimed to present our experience about the use of colistin in severe nosocomial infections in our PICU during a longer period of time and with newly emerged patients.

A few studies have investigated the efficacy and safety of colistin therapy in children thus far, and the majority of existing studies have been performed in specific patient groups such as newborns and patients with cystic fibrosis or burn injuries [4,11,17]. No special patient group was singled out in our study, differentiating it from previous studies. The results of present study have suggests that colistin may be a viable option in the treatment of serious pediatric nosocomial infections caused by MDR gram-negative bacteria.

The optimum pediatric dosing for colistin is not clear due to lack of the pharmacokinetic and pharmacodynamic studies in this age group. However, an intravenous dose of 2.5-5 mg/kg/day has been recommended [2,4,11,18]. In our study we found the average dose of 4.9 mg/kg/day divided into 2 or 3 equal doses effective and safe.

The major side effects of treatment with colistin are neurotoxicity and nephrotoxicity [4,8,11,12,19]. Recent pediatric studies have reported a nephrotoxicity rate of 0–14.3% [4,8,10-12,18]. This rate is lower than that given in previously reported results in adults (5.8-26.8%) [20-22]. The main mechanism of renal injury is acute tubular necrosis. Renal injury usually occurs in the early stages of treatment and characterized by a decrease in creatinine clearance and increase in serum urea and creatinine levels [2,17]. In our study renal toxicity occurred at the median 8th day of treatment (relatively late). Less commonly, hematuria, proteinuria, and cylindruria may also be seen [17]. Similar to our study sepsis, hypotension and the use of other nephrotoxic drugs due to nosocomial infections may potentiate the nephrotoxicty of colistin [4,10,12,17]. Many studies have reported that the use of other nephrotoxic drugs such as aminoglycosides together with colistin facilitates the deterioration of renal function [4,10,12,17,23-26]. In our study colistin was used with other nephrotoxic agents such as aminoglycoside, vancomicyn or amphotericin B in twenty-nine of forty-one episodes of nosocomial infection and only three patients developed nephrotoxicity. Similarly, Jajoo and colleagues [17] reported that nephrotoxicity occured in only one of six newborns after the administration of netilmicin. Interestingly, Li and colleagues [25] reported that colistin is less nephrotoxic than amikacin or tobramycin. Both adult and pediatric studies have reported that the nephrotoxicity of colistin is reversible and renal function returns to normal with discontinuation of treatment [2,8,19]. In our study treatment with gentamicin was halted in one patient because of nephrotoxicity, but colistin therapy was continued due to life-threatening sepsis. With peritoneal dialysis, renal function returned to normal levels 18 days after the end of colistin treatment in this patient. In other patient, colistin treatment was changed with meropenem and renal function returned to normal levels within days. In our unit, none of the patiens used colistin developed nefhrotoxicity in the last two years. None of the patient used colistin in the last two years developed nephrotoxicity. We attribute this to the increased sensitivity of our team regarding renal toxicity, to closer monitoring of fluid balance and renal functions in colistin users, and to the avoidance of the use of concomitant nephrotoxic agents.

There are very few reported neurological side effects of colsitin in pediatric studies [8,11,12,17], and neurological side effects were not observed in our study. The challenge of detecting neurological side effects in patients with underlying neurological disease may be the reason for this. Other possible reasons include the fact that drugs frequently used in the intensive care unit (such as sedative/analgesic drugs, or neuromuscular blocking agents) can mask neurological side effects and neurological side effects may also be overlooked in retrospective studies. Finally, the use of sodium colistimethate (which is less toxic than colistin) in our study may be another reason for the lack of neurological side effects [8,16].

Favorable clinical response rates have reportedly been higher in pediatric studies than in adult studies (76–86.5% versus 57-73%, respectively) [8-10,20,27]. A favorable outcome occurred in 68.3% courses and was consistent with the previous studies. In addition, positive clinical and microbiological response was achieved in 6 out of 7 episodes developed in 4 patients with primary immune deficiency.

The synergistic effect of concomitant use of colistin with rifampicin, carbapenems, piperacillin/tazobactam, or ciprofloxacin has been reported in previous studies [24,25,28-32]. Similar to previous studies, we used colistin in various combinations with other antimicrobial agents. In this study, colistin was combined with an antibiotic against on gram-negative bacteria in almost all cases. We believe that combination regimen can contribute to the success rate of treatment.

Prognosis in the case of a bacterial infection caused by MDR is affected by the presence of underlying disease, sepsis, or organ failure. Of the 35 patients in our study, 30 had underlying chronic diseases and 4 had immunodeficiency. A mortality rate of 2-28% has been reported in previous studies [8,11,12,17]. Our results are similar to previous reports. While 6 out of the 12 patients who died during the study period died of infection-related reasons, the others died because of their primary diseases such as multiple trauma, malignancy and congenital heart diseases.

The major limitations of this study include the retrospective design and the insufficient number of patients which is too limited to determine the true incidence of drug-related side effects. Additionally, patients had taken many drugs before and during treatment with colistin. Therefore, it was not possible to associate all the effects and side effects that occurred during the study with colistin alone.

In conclusion, the results of this study have shown that colistin is effective in the treatment of serious nosocomial infections caused by MDR gram-negative bacteria in children. Renal function test must be closely monitored during the treatment period. We suggest avoiding the use of other nephrotoxic drugs with colistin to minimize the nephrotoxicity. However, more prospective controlled trials are needed to objectively evaluate the efficacy and side effects of this treatment.

## Competing interests

The authors declare that they have no competing interests.

## Authors’ contributions

AK: wrote the manuscript. MSP: took care of the patients in the PICU, supervised the work and corrected the manuscript for publication. AK: microbiologist of the study. NB and SGS: infectious disease consultant of the patients. SP: reviewed the patient charts and prepared the manuscript. AKG: performed the experiments, analyzed the results. MA: reviewed the patient charts. NY: took care of the patients in the PICU. All authors read and approved the final manuscript.
